# Editorial: Emergent Neural Computation from the Interaction of Different Forms of Plasticity

**DOI:** 10.3389/fncom.2015.00145

**Published:** 2015-11-30

**Authors:** Matthieu Gilson, Cristina Savin, Friedemann Zenke

**Affiliations:** ^1^Computational Neuroscience Group, Department of Technology and Information of Communication, Universitat Pompeu FabraBarcelona, Spain; ^2^Institute of Science and Technology AustriaKlosterneuburg, Austria; ^3^Neural Dynamics and Computation Lab, Department of Applied Physics, Stanford UniversityStanford, CA, USA

**Keywords:** homeostatic plasticity, inhibitory plasticity, learning, neural network, neuromodulation

More than 60 years later, Hebb's prophecy “neurons that fire together wire together” (Hebb, 1949; Shatz, 1992) prevails as one of the cornerstones of modern neuroscience. Nonetheless, it is becoming increasingly evident that there is more to neural plasticity than the strengthening of synapses between co-active neurons. Experiments have revealed a plethora of synaptic and cellular plasticity mechanisms acting simultaneously in neural circuits. How such diverse forms of plasticity collectively give rise to neural computation remains poorly understood. The present Research Topic approaches this question by bringing together recent advances in the modeling of different forms of synaptic and neuronal plasticity. Taken together, these studies argue that the concerted interaction of diverse forms of plasticity is critical for circuit formation and function.

A first insight from this Research Topic underscores the importance of the time scale of homeostatic plasticity to avoid runaway dynamics of Hebbian plasticity. While known homeostatic processes act slowly, on the timescale of hours to days, existing theoretical models invariably use *fast homeostasis*. Yger and Gilson (2015) review a body of theoretical work arguing that rapid forms of homeostatic control are in fact critical for stable learning and thus should also exist in biological circuits. Following a similar line of thought, Chistiakova et al. (2015) review experimental and theoretical literature which suggests that the role of rapid homeostasis could be filled by heterosynaptic plasticity. Alternatively, other mechanisms can achieve a similar stabilizing effect, as long as they are fast, for instance the rapid homeostatic sliding threshold in Guise et al. (2015). These findings raise questions concerning the purpose of slow homeostasis and metaplasticity. Since non-modulated plasticity leads to “interference” between memories when confronted with rich environmental stimuli (Chrol-Cannon and Jin, 2015), it is tempting to hypothesize that certain slow homeostatic mechanisms may correct for this (Yger and Gilson, 2015).

The second development reflected in this Research Topic concerns the interactions between *excitatory and inhibitory* (E/I) plasticity. Multiple studies independently stress the importance of such interactions for shaping circuit selectivity and decorrelating network activity during learning. Kleberg et al. (2014) demonstrate how spike-timing-dependent plasticity at excitatory (eSTDP) and inhibitory (iSTDP) synapses drives the formation of selective signaling pathways in feed-forward networks. Together they ensure excitatory-inhibitory balance and sharpen neuronal responses to salient inputs. Moreover, by systematically exploring different iSTDP windows, the authors show that anti-symmetric plasticity, in which pre-post spike pairs lead to potentiation of an inhibitory synapse, are most efficient at establishing pathway-specific balance. Zheng and Triesch (2014) confirm the relevance of e/iSTDP for propagating information in a recurrent network. Their model also highlights the importance of other forms of plasticity, in particular *intrinsic plasticity* and *structural plasticity* for robust synfire-chain learning.

Beyond information propagation, Duarte and Morrison (2014) show that E/I plasticity allows recurrent neural networks to form internal representations of the external world and to perform non-linear computations with them. They find that the decorrelating action of inhibitory plasticity pushes the network away from states with poor discriminability. These results are corroborated by Srinivasa and Cho (2014), who show that such representations can be efficiently picked up by downstream layers. Networks shaped by both e- and iSTDP learn to discriminate between neural activity patterns in a self-organized fashion, whereas networks with only one form of plasticity perform worse. Binas et al. (2014) show that the interplay of E/I plasticity in recurrent neural networks can form robust winner-take-all (WTA) circuits, important for solving a range of behaviorally relevant tasks (e.g., categorization or decision making). Using a novel mean-field theory for network dynamics and plasticity, the authors characterize parameter regions in which stable WTA circuits emerge autonomously through the interaction of E/I plasticity.

While most work presented here focuses on long-term plasticity, Esposito et al. (2015), study the interactions between Hebbian and *short-term plasticity* (STP) at excitatory synapses. The authors postulate a form of *metaplasticity* that adjusts the properties of STP to minimize circuit error. This model provides a normative interpretation for experimentally observed variability in STP properties across neural circuits and its close link to network connectivity motifs. While detailed error computation as assumed here is biologically implausible, reward-related information could be provided by neuromodulators (in particular, dopamine), which are know to regulate circuit dynamics and plasticity.

The functional importance of *neuromodulation* is explored in two papers. First, Aswolinskiy and Pipa (2015) systematically compare reward-dependent vs. supervised and unsupervised learning across a broad range of tasks. They find that, when combined with suitable homeostatic plasticity mechanisms, reward-dependent synaptic plasticity can yield a performance similar to abstract supervised learning. Second, Savin and Triesch (2014) use a similar circuit model to study how reward-dependent learning shapes random recurrent networks into working memory circuits. They show that the interaction between dopamine-modulated STDP and homeostatic plasticity is sufficient to explain a broad range of experimental findings regarding the coding properties of neurons in prefrontal circuits. More generally, these results enforce the idea that reward-dependent learning is critical for shifting the limited neural resources toward the computations that matter most in terms of behavioral outcomes.

Taken together, the contributions to this Research Topic suggest that circuit-level function emerges from the complex, but well-orchestrated interplay of different forms of neural plasticity. To learn how neuronal circuits self-organize and how computation emerges in the brain it is therefore vital to focus on interacting forms of plasticity. This sets the scene for exciting future research in both theoretical and experimental neuroscience.

## Funding

CS acknowledges funding from the People Programme (Marie Curie Actions) of the European Union's Seventh Framework Programme (FP7/2007-2013) under REA grant agreement n° [291734]. MG acknowledges funding from the Human Brain Project (PR10513 :EC-7PM-HBP). FZ was supported by the SNSF (Swiss National Science Foundation) Mobility fellowship P2ELP3_161836.

### Conflict of interest statement

The authors declare that the research was conducted in the absence of any commercial or financial relationships that could be construed as a potential conflict of interest.
